# The steep road to nonviral nanomedicines: Frequent challenges and culprits in designing nanoparticles for gene therapy

**DOI:** 10.3762/bjnano.14.30

**Published:** 2023-03-17

**Authors:** Yao Yao, Yeongun Ko, Grant Grasman, Jeffery E Raymond, Joerg Lahann

**Affiliations:** 1 Biointerfaces Institute, University of Michigan, Ann Arbor, MI 48109, USA; 2 School of Dentistry, University of Michigan, Ann Arbor, MI 48109, USA; 3 Department of Chemical Engineering, University of Michigan, Ann Arbor, MI 48109, USA; 4 School of Polymer Science and Engineering, Chonnam National University, Buk-gu, Gwangju 61186, South Korea

**Keywords:** characterization, dosage, gene delivery, uptake, transfection

## Abstract

The potential of therapeutically loaded nanoparticles (NPs) has been successfully demonstrated during the last decade, with NP-mediated nonviral gene delivery gathering significant attention as highlighted by the broad clinical acceptance of mRNA-based COVID-19 vaccines. A significant barrier to progress in this emerging area is the wild variability of approaches reported in published literature regarding nanoparticle characterizations. Here, we provide a brief overview of the current status and outline important concerns regarding the need for standardized protocols to evaluate NP uptake, NP transfection efficacy, drug dose determination, and variability of nonviral gene delivery systems. Based on these concerns, we propose wide adherence to multimodal, multiparameter, and multistudy analysis of NP systems. Adoption of these proposed approaches will ensure improved transparency, provide a better basis for interlaboratory comparisons, and will simplify judging the significance of new findings in a broader context, all critical requirements for advancing the field of nonviral gene delivery.

## Introduction

Recent efforts to develop and translate therapeutically loaded nanoparticles (NPs) have resulted in several advances in the treatment and prevention of disease. Key areas where NP materials have had an impact include their use as vaccines, cancer therapies, and in the treatment of rare genetic disorders [1–3]. Still, there are several impediments to characterizing, understanding, and controlling the interactions between NPs and biological substrates [4–6].

Standardization of NP characterization is a widely recognized issue that is critically entwined with scientific and technical considerations [7–8]. In fact, the importance of standardization within the field of nanomaterials has already been highlighted in previous editorials and perspectives, illuminated by “Nature Nanotechnology”’s editorial that “few studies offer consistent results that are of value, and it is difficult to compare studies because they are often carried out using poorly characterized nanomaterials and arbitrary experimental conditions” [9]. In addition to the difficulty of interexperimental and interlaboratory comparisons, there is also significant uncertainty and ambiguity in drawing conclusions from results that were obtained by fundamentally different study designs. A wide spectrum of scientific approaches as well as variable strengths and weakness associated with each of these characterization methods, de facto makes a direct comparison between studies impossible. In addition, the area of nanomedicine is plagued by the tendency of researchers to use various (often arbitrarily defined) reference materials, different techniques for dosage selection and determination, and assess outcomes and efficacy against various cell types using differing characterization methods and metrics [8]. This divergence of methods and analytical practices severely hinders the evaluation of new NP-based therapies and constrains the unbiased evaluation of new findings by the nanomedicine community. Therefore, best-practice standards for high-quality NP studies are urgently needed [4–5].

Recommendations from the community to address these issues exist and include encouraging the use of multiple characterization techniques, clarifying focused research questions [10], and performing material or biological characterizations suitable to the specific type of investigation [11]. Among these recommendations, “MIRIBEL” (Minimum Information Reporting in Bio-Nano Experimental Literature) was proposed in 2018 for the community to adopt a “reporting standard” to enhance the quality and reproducibility of published research [6]. MIRIBEL champions four guiding principles, namely reusability, quantification, practicality, and quality.

In nanomedicine, these challenges are further compounded by the fact that the study of NP interactions with biological systems is a multidisciplinary field that ranges from understanding fundamental biological interactions [12] to engineering nanomaterials for specific applications [13–14]. One particular topic of recent interest is NP-mediated nonviral gene delivery, which is the focal point of this perspective. Emerging NP-mediated nonviral gene delivery systems have gathered significant attention due to the COVID-19 pandemic and the breathtaking acceptance of mRNA-based vaccines [15].

Following the spirit of MIRIBEL, the current perspective was written with two specific aims in mind. First is to provide an overview of current concerns regarding aspects of NP uptake, transfection efficiencies, payload retention, and interparticle variability in the context of nucleic acid-based nanotherapeutics. In order to illustrate these issues and concerns, we analyzed the materials and methods sections of 50 papers published within the last five years on the topic of NP-mediated delivery of plasmid DNA after reasonable search and selection. Secondly, our objective is to emphasize the importance of standardized practices for the investigation of NP dosing, uptake, and transfection to improve the reusability, quantification, practicality and quality in the field of nonviral gene delivery research. Of note, this perspective is not intended to provide a comprehensive overview of the best practice (for such comprehensive reviews the reader is referred to for instance Rennick et al. [1]), but rather aims at highlighting the importance of multimodal, multiparameter, and multistudy analysis of NP systems based on specific issues. In addition, we considered only plasmid-based transfection systems during literature search (see details about experimental approach and scope in Supporting Information File 1). But we would expect a similar outcome and trend for the mRNA or siRNA NP delivery literature.

## The Need for Multimodal Characterization of Nanoparticles

The methods chosen to investigate NP uptake and transfection can be biased towards particular properties and may provide limited insights into the efficiency of NP internalization and efficacy. Typically, cellular uptake and transfection efficiency are assessed with the use of fluorescent-labeling carriers and the expression of fluorescent proteins (e.g., enhanced green fluorescent protein). Both of which are typically assessed by widefield fluorescent microscopy/confocal microscopy (referred to as “imaging”) and/or flow cytometry (Table 1).

**Table 1:** Characteristics of techniques used to investigate cellular uptake and transfection of NPs.

Advantages	Disadvantages

Widefield fluorescence microscopy/confocal microscopy	

+ provides information of NP spatial distributions, aggregations and NP-cell interactions; + time-lapse live cell imaging [16]: (i) reveals NP dynamics and fate; (ii) reveals the effects of NPs on the recipient cells in real-time; (iii) allows for multiple timepoints to be observed with one experiment; (iv) prevents possible artifacts induced by the fixation of cells prior to microscopic examination	− low throughput (especially high-resolution imaging and 3D imaging capture) [1]; − ambiguous when determining internalization within 500 nm of cell membrane [17]; − 2D images inaccurate for counting of NPs or organelles ; − subjective interpretation; − requires dedicated imaging set-up for live cell imaging

Flow cytometry	

+ high throughput (rapid acquisition of large data sets); + analysis at single-cell level; + more than 20 wavelength channels available	− cannot distinguish internalized NPs from surface-bound NPs (extra step needed) [17]; − no information about the location of fluorescent signals.

Imaging cytometry	

+ combines the high-event-rate nature of flow cytometry with the advantages of single-cell image acquisition associated with microscopy [18]; + enables the recognition and enumeration of subcellular areas	− lower resolution than confocal microscopy; − unable to generate 3D cell imaging reconstruction (only acquire a single ca. 1µm thick cross-sectional image of the cell) [17]; − lacking capability for workflow automation and cell sorting; − trade-off between the acquisition speed and image quality.

**Figure 1:**
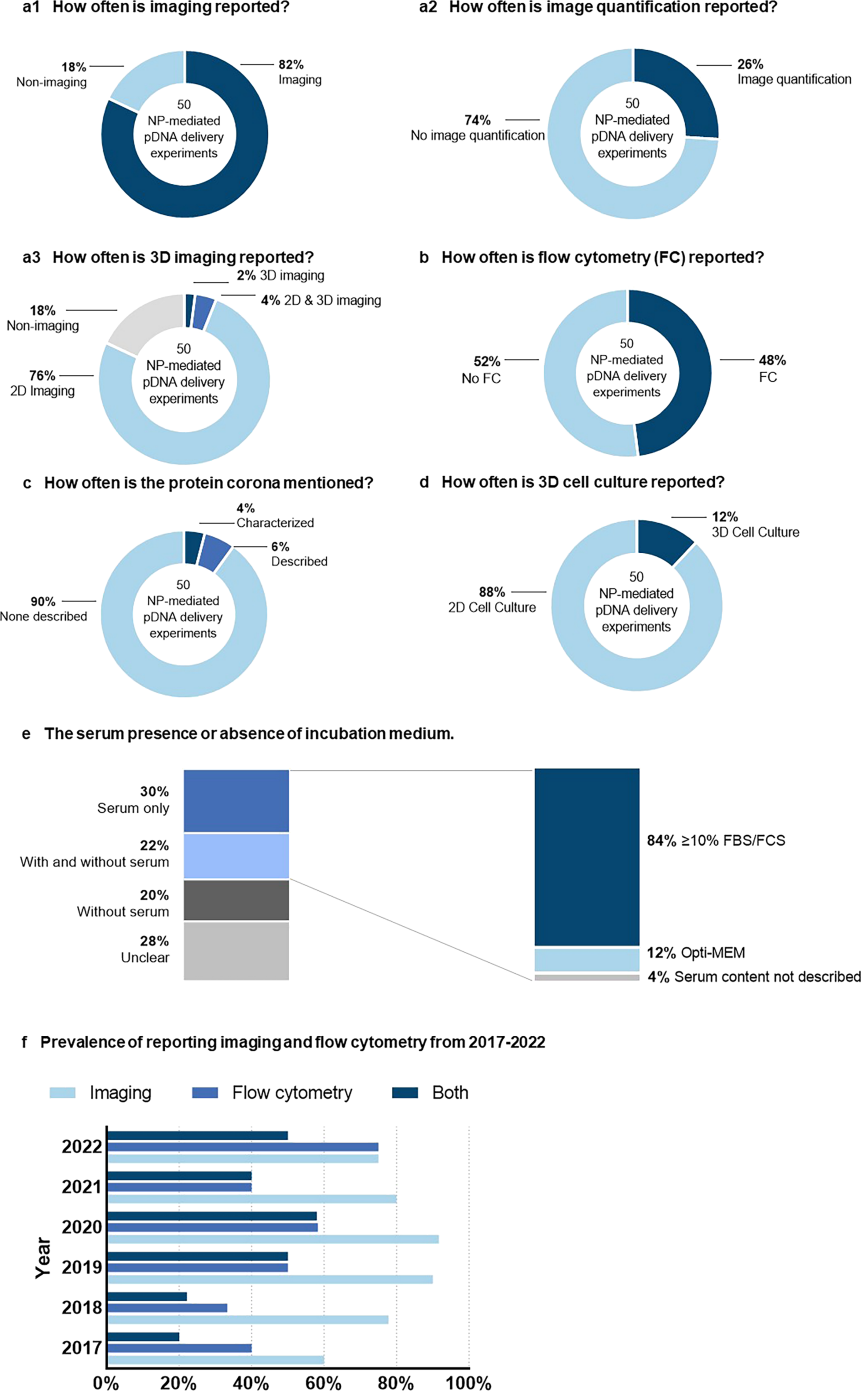
Prevalence of reporting imaging and (or) flow cytometry techniques, protein corona, 3D cell culture model(s), and serum content during nanoparticle incubation with cells in 50 nanoparticle (NP)-mediated plasmid DNA (pDNA) delivery experiments published in 2017–2022. (a) 5-year prevalence of reporting imaging, imaging quantification, and 3D imaging capture for investigating NP cellular uptake and (or) transfection. (b) 5-year prevalence of reporting flow cytometry for investigating NP cellular uptake and (or) transfection. (c) 5-year prevalence of describing or characterizing protein corona in the manuscript. (d) 5-year prevalence of employing 3D cell culture models (e.g., spheroids or organoids). (e) Comparisons of serum content used during nanoparticle incubation with cells. (f) Annual prevalence of reporting imaging and (or) flow cytometry during the last five years. Of note, “imaging” refers to images captured by widefield fluorescence microscopy or confocal microscopy (electron microscopy excluded). See details about experimental approach and scope in Supporting Information File 1.

Laser scanning or spinning disc confocal microscopy, typically used for qualitative determination of NP uptake, can provide valuable insights into relative distribution and aggregation of NPs, which has been widely used in the field of nonviral gene delivery (reported in 82% of the papers, Figure 1a) [16]. Unfortunately, confocal imaging is limited by relatively low throughput (even with automation) and can be ambiguous when determining internalization within 500 nm of the cell membrane [17]. However, widefield fluorescence microscopy is still widely used when it comes to observing the expression of proteins after transfection. Because organelles (e.g., endosomes) are not uniformly distributed throughout the cell, these 2D imaging methods are rarely accurate for quantification of either uptake or transfection. Nevertheless, they still have been the methods of choice during the last five years (only 6% used 3D imaging, Figure 1a3) [19]. Furthermore, the subjective interpretation of results obtained by imaging techniques cannot be neglected [17]. Based on 50 studies regarding NP-mediated delivery of plasmid DNA to cells/tissues, only a small fraction reported systematic image quantification after image capture (26%, Figure 1a2).

Flow cytometry, also a fluorescence-based method, is an alternate assessment of uptake and transfection. It is commonly used for high-throughput cell studies and is known for both rapid data acquisition and large data sets [17]. Flow cytometry can generate large data sets at rates greater than one thousand cells per second [20]. This allows for a more robust statistical analysis resulting in a greater degree of confidence. In spite of its clear merits, this technique has not been accepted by the community as much as imaging (reported in less than 50% of the papers, Figure 1b). Despite the robust data it can provide, flow cytometry counts the total number of cells associated with NPs and cannot distinguish internalized cargo from surface-bound NPs [1,17]. To precisely quantify internalization, a secondary method is required that can differentiate between surface-bound and internalized NPs. Commonly used techniques are far from optimal, but include non-specific methods for removing surface-bound NPs or quenching surface-bound fluorescence [17]. In addition to the distinction between membrane binding and internalization, there are other concerns associated with flow cytometry. As more researchers prefer to use mean fluorescence intensity (MFI) to determine the uptake or transfection of cells, an important metric, the percent positive value, is often omitted. It is important to report both percent positive values and MFI, as they provide complementary information about an experiment [17]. This is true for both NP uptake studies and transfection readouts. In regard to NP uptake, reporting percent positive values makes it possible to discern whether subpopulation of cells with a high number of NPs per cell exist or if a lesser number of NPs is taken up by a large fractions of cells. Similarly, when assessing transfection, it is important to know if a small population of cells with high expression profiles exists or if large fractions of cells with lower levels of transfection dominate the population [8]. A detailed analysis of these values and their distributions can provide critical insights into the mechanisms of uptake and transfection.

Alternatively, imaging flow cytometry combines the high-throughput nature of flow cytometry with a high degree of spatial resolution and has been recently developed and employed in nonviral gene delivery research [21]. This technology measures the mean fluorescence intensity and percent positive value of cells, while also capturing an image of each individual event; this provides information, such as cellular distribution patterns of NPs [22], while also allowing for investigating cellular uptake pathways and intracellular trafficking [23]. In the absence of imaging flow cytometry, a two-pronged approach (high-resolution imaging plus a separate high-throughput analysis) is recommended to ensure the highest confidence in the result. Our literature assessment also revealed an increasing preference among the community for employing both imaging and flow cytometry for the investigation of NP uptake and transfection (Figure 1f). Specifically, there is a clear benefit in reporting a variety of values when disclosing fluorescence-based results (e.g., MFI, intensity histogram, and percent positive values).

## Uptake Mechanism

Currently, extensive research is focused on the improvement of NP carrier design and gene delivery efficiency [23–26]. Recent debates within the gene delivery field have highlighted the need for an improved understanding about what cellular/subcellular features underscore the success of one formulation or the failure of another [15,17,27–28].

Within this context, an essential question is: “how are NPs internalized by cells?” [29]. Several mechanisms of endocytosis have been identified as listed in Table 2 [1]. Conventionally, the significance of an endocytosis pathway for a particular type of NP can be measured using pharmacological inhibitors or genetic approaches that knockdown/knockout or transiently block the expression of key proteins involved in endocytosis [30]. Then, changes in NP uptake can be quantified and attributed to the significance of that pathway. Unfortunately, upon limiting the function of a certain endocytosis pathway, especially through the use of pharmacological inhibitors, cells can respond to the blockage by overactivating alternative mechanisms that would normally be less relevant [31]. This makes the interpretation of each pathway’s significance more complicated and sometimes ambiguous [32]. To circumvent these issues, given the advancement and prevalence of high- or super-resolution microscopy, imaging-based approaches can be used to directly visualize uptake and determine whether the NP is co-localized or associated with key endocytic structures or proteins [33–34].

**Table 2:** Toolbox of pharmacological inhibitors used to study endocytosis pathways.

Pathways	Commonly used pharmacological inhibitors	Mechanism of inhibitors	Potential pitfall of inhibitors	Conc. range

macropinocytosis	amiloride and its derivatives 5-(*N*-ethyl-*N*-isopropyl) amiloride (EIPA)	inhibits Na^+^ channels and Na^+^/H^+^ exchange	(i) has been shown to inhibit FEME^a^; (ii) may affect actin [37]	1 mM for amiloride and 25-100 μM for EIPA [30,33]
CME^b^	chloropromazine	disrupts clathrin and the AP2 complex from the cell surface	inhibits FEME, not efficient in all cell lines, affects biogenesis of large intracellular vesicles such as phagosomes and macropinosomes	30–60 μM [30,38]
caveolae	methyl-β-cyclodextrin	removes cholesterol from the plasma membrane	(i) interferes with other uptake mechanisms [39] including macropinocytosis and CME because of changes in membrane fluidity; (ii) affects actin cytoskeleton [40]	2.5–10 mM [30,41–42]
	filipin III	interacts with cholesterol at the cell membrane	(i) permeabilization of the plasma membrane occurs; (ii) involves disruption of the linkages between F-actin and plasma membrane [35]	1–5 μg/mL [43–46]

^a^FEME: clathrin-independent/dynamin-dependent endocytosis; ^b^CME: clathrin-mediated endocytosis.

Overall, there is still a strong tendency for researchers to select pharmacological inhibition over genetic studies. This is due to several considerations, namely (i) rapid action in blocking the uptake route, (ii) equal inhibition of the overall cell population, and (iii) time- and labor-efficient processing [29,35]. However, it is well known that pharmacological inhibitors have severe limitations. These include varying levels of specificity (Table 2), significant cytotoxicity, low selectivity, and variable efficacy that can change within different cells lines and different experimental setups [1,30,35]. The proper use of transport inhibitors requires stringent controls to substantiate their effects and to rule out artifacts. Typically, this requires the use of appropriate markers that have been extensively validated to be specifically internalized by particular pathways. Transferrin [1] can be used as a maker for clathrin-mediated endocytosis (CME), bodipy-lactosylceramide (LacCer) can be used for caveolae-mediated endocytosis, and dextran with large molecular masses can be used for macropinocytosis [36]. Moreover, concentration should be optimized (Table 2) and toxicity should be closely monitored, as cell death can be misinterpreted as efficient inhibition, especially in metabolism-based assays. For these inhibitors that involve the permeablization of plasma membranes, such as filipin III, appropriate controls for plasma membrane integrity during the inhibition exposure should be included [35].

In order to overcome the poor specificity of pharmacological inhibitors, genetic approaches can be implemented to change the expression of specific proteins [29]. However, the complexity and care with which these studies must be undertaken are higher than an inhibition approach. One genetic approach is the knockout/knockdown of key components in internalization pathways [47]. Alternatively, the expression of dominant-negative inhibitors may be used [48]. If performed carefully with appropriate controls, both can largely overcome major problems associated with pharmacological inhibitors. However, genetic alterations may also result in changes that share protein components or lead to compensatory mechanisms in the cell [37]. Despite their intrinsic specificity, validation still remains critical to avoid affecting multiple pathways. Hence, the inclusion of appropriate positive and negative controls is crucial to avoid misinterpretations if genetic approaches are used.

In addition to (or in tandem with) inhibition and genetic strategies, imaging-based approaches should be performed. These imaging studies aid in the identification of the internalization mechanisms via fluorescence co-localization analysis of endocytic biological markers and NPs [33]. While co-localization studies by means of overexpression of fluorescent proteins of key endocytic regulators or immunostaining for these regulators can be helpful in determining endocytosis routes of NP uptake, care should be taken to prevent artifacts [49]. New biological tools, such as SNAP-tag, could be used to label intracellular proteins with high efficiency and low fluorescence background, which would be promising for future co-localization studies investigating NP-mediated endocytic routes [50–51].

The combination of multiple methods among those available (each presenting advantages and limits) is probably the best approach to try to fully characterize and investigate the pathways involved and answer essential questions. An idealized scenario may be one in which inhibition results are coupled to both knockdown and imaging data to provide a full understanding of the mode of action in NP uptake studies.

## Determination of In-Particle Plasmid Dosage

There are a variety of approaches for the evaluation of dose in the context of small-molecule therapeutics encapsulated in NP delivery systems [52–54]. However, many of the practices used to determine small-molecule dosing become confounded if applied to nucleic acid payloads (such as plasmids). Direct mass determination of the internalized nucleic acid therapeutics (NATs) is not straightforward and is further complicated given that dosing in these systems should be considered in multiple ways. We recommend considering mass concentration (both NATs and NPs), number concentration (NPs/mL), and NP potency (molecules of NAT/NPs) [55].

**Figure 2:**
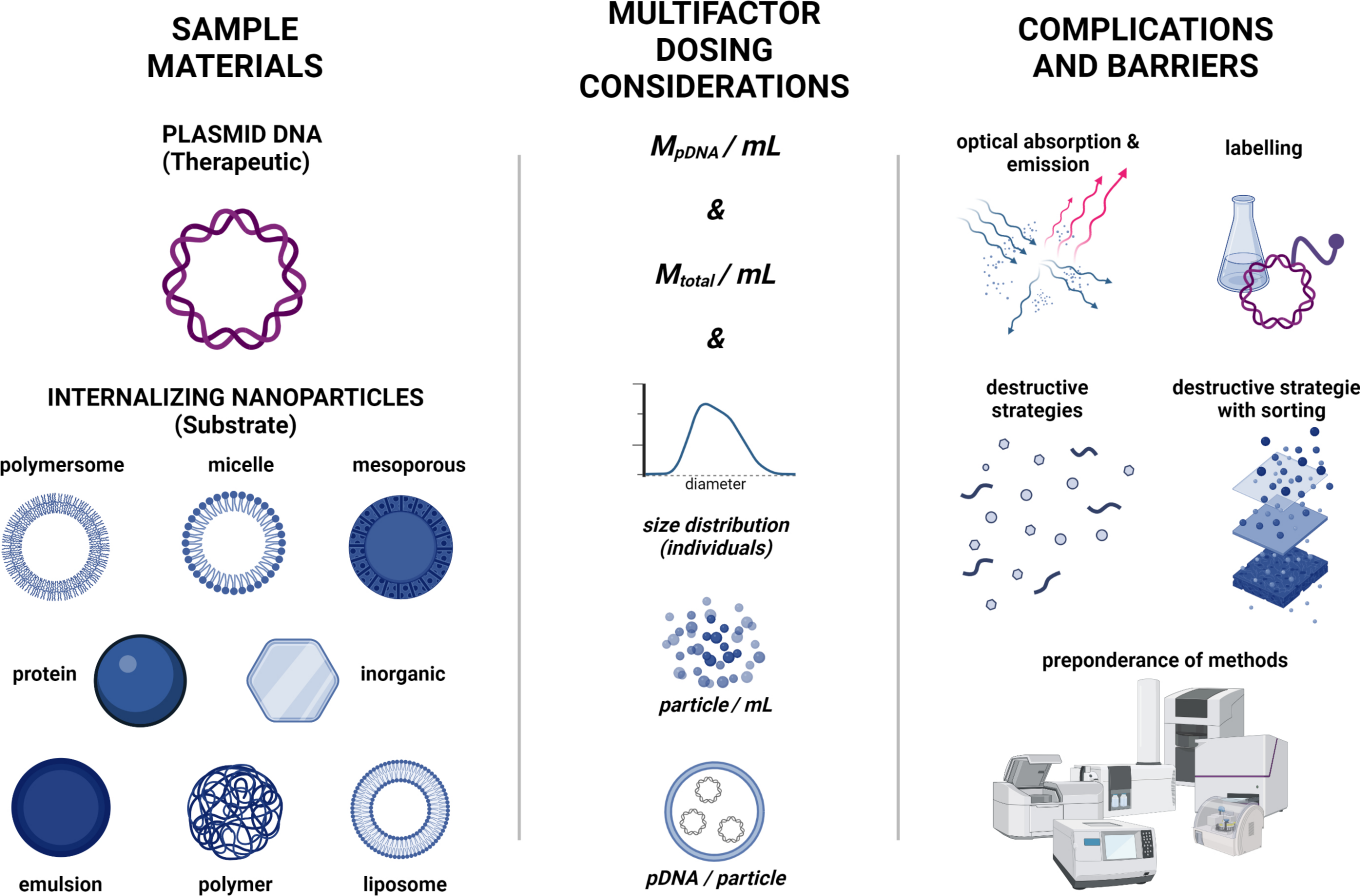
Practical concerns regarding dose determination in nucleic acid–NP systems. Left: Examples of possible pDNA internalization regimes. Center: Recommended dosing metrics that would be helpful for comparison between systems. Right: Barriers to acquisition of all key dosing metrics. Created with BioRender.com (https://biorender.com/). This content is not subject to CC BY 4.0.

Conventional colorimetric or fluorescence assays are insufficient for the assessment of NAT-NP systems. These assays often rely on small to mid-size molecular probes that bind and associate nonspecifically (structural adsorption, intercalation, or backbone interactions) or through explicit interactions with specific NAT residues [56]. Ultimately, the particulate structure of these systems can result in transport constraints into and within the nanoparticle and, thus, in preventing the diffusion and association of probe molecules [6]. For these types of assays, it is very common for the result of concentration measurements to be significantly lower than those obtained from free NAT systems with identical concentrations. Inclusion of polymeric (e.g., core–shell micelles), oligomeric (e.g., lipid nanoparticles), or biomacromolecular (e.g., protein nanoparticles) components complicates matters only further by generating a higher-than-normal background through non-specific interactions with the assay media. In addition, a significant bias may arise from strong associations between carrier and payload [57]. Without careful deconvolution, the scattering signal from a particulate analyte can readily result in a high bias that compromises UV–vis-based analyses. Ambiguity can further be enhanced in fluorescence assays because of the drastically different environments that can impact quantum yield [58]. These complications, along with others, are highlighted in Figure 2.

Several alternate options exist that can provide reasonable assessment of NAT in NP systems. Firstly, NAT-labeling strategies beyond fluorescent labels, such as radiolabeling and elemental labeling have been explored [59]. However, these approaches require chemical modification of the payload, which is often not desired. In some cases, disassembly of the NP prior to analysis may also be possible, with a subsequent use of colorimetric assays. The difficulty with this approach is that accounting for substrate contributions or proving the full availability of the NAT can be difficult and laborious. Alternatively, chromatographic and size-exclusion techniques, elemental analysis, or polymerase chain reaction (PCR) [60] mass spectrometry [61] can be effective if the payload can be separated from the NP prior to analysis.

Regardless of the approach, it is worth noting that NAT concentration alone cannot address the extent of loading (i.e., the NAT/NP mass ratio) or therapeutic efficacy (e.g., plasmids/particle). Nanoparticle tracking analysis (NTA) [62] can provide insights into the concentration, while indirect interconversion of size distribution and concentration to mass can provide a reasonable non-destructive path to the assessment of dose based on total particle mass. The latter allows the system to be assessed on a per-particle basis with subsequent determination of particle potency (NAT per NP). Other multiexperiment options, though time-consuming, may allow for the proper level of NP characterization. For example, the use of multi-angle light scattering [63], gravimetric analysis, and particle counting, if used in a concerted approach, can provide size distribution, mass-based concentration, and particle-based concentration information. Together, with proper NAT determination, such multimodal design will allow for determination of all critical dosing metrics as discussed above.

These considerations, when taken together, highlight the need for advanced analytical tools that are reproducible, accurate, and non-destructive in the assessment of NP-encapsulated NAT payloads (mass/volume, mass/mass, and on a per-particle basis).

## Interparticle Dose Variability

In the previous discussion, an assumption of particle dose scaling with particle mass is made. However, in the case of large molecules (such as plasmids) in smaller particles, significant deviations from this trend can be observed. To better understand the per-particle variations in payload, high-resolution microscopy, such as total internal reflection fluorescence spectroscopy, near-field scanning optical microscopy, or super-resolution microscopy (e.g., STORM, SIM) can be used to establish relative emission ratios for a labeled payload relative to a labeled carrier.

### Regarding standard methods

Given the number of possible techniques for the assessment of the size distributions of NP systems, we offer some standards on the most commonly found techniques with an intent to provide a starting point for the development of repeatable protocols.

#### Imaging (individual analysis)

For particles that are readily measured in the solid state, we recommend the ISO 19749:221 standard for SEM analysis, ASTM E2859-11 for AFM analysis, and ISO 21363:2020 for substrate-supported TEM analysis. When at all possible, obtaining the distribution of sizes from these methods with extension to obtain geometric properties (aspect ratio, minimum diameter, circularity, roundness, or sphericity) will allow for the comparison within different batches, formulations, particle types, and standards. For particle systems that do not exist in a solid state, cryo-TEM or submersion AFM can offer alternative routes. These measurements will provide high-resolution particle distributions for in-solvent particles, though they may not be as definitive as the solid-state particle distributions.

#### Mobile solution phase particle size distributions (individual and ensemble analysis)

In most instances, it will be valuable to assess the hydrodynamic size of the NPs. First, a quantification of the central tendency of the diameter or radius (e.g., mean, mode, median, or intensity maxima) should be reported. Additionally, a presentation of population variance (e.g., standard deviation for normal distributions, transform standard deviation for lognormal distributions, range interquartile ranges, 5–95 band, or 10–90 band) will be of use, specifically for dose/particle calculations. Lastly, a volume or mass distribution should be presented. Dynamic light scattering (DLS) is often be used for the inspection of aqueous suspensions with results bedeviled by the normal constraints of DLS and ensemble detection [64]. Both disc centrifugation and ultracentrifugation provide ensemble detection of size distributions with their own issues and complexities (e.g., gradient-induced aggregation and pressure-induced particle reconfiguration) [65]. Determination of size on a per-particle basis, such as that obtained from NTA or the aforementioned cryo-TEM/solution AFM will be critical to obtaining detailed dosing information [66]. While tunable resistive pulse sensing will also provide similar information, it is less utilized and less understood than the methods listed above [67]. As a final note, it is also recommended that both an “as used” solvent and a reference solvent are used (e.g., deionized/polished deionized water or common buffer) allowing for comparison to other studies. Regarding standard practices, ASTM E3247 provides excellent advice for the assessment of monodisperse samples by DLS with a focus of accuracy, repeatability, reproducibility, and bias in measurement. For NTA, ASTM 2834 provides workflows for planning NP experiments. Through the study of nanoscale extracellular vesicles, Bachurski et al. [68] provide some additional insights comparing the performance of different NTA systems to cryo-TEM and single-particle interferometric reflectance imaging sensing methods.

## Future Directions and Outlook

- Common techniques used to determine uptake and transfection possess limitations. Image-based techniques, such as widefield fluorescence microscopy and confocal microscopy, are limited by relatively low throughput and can be ambiguous for properly quantifying NP internalization by cells. Flow cytometry allows for high-throughput analysis of thousands of cells but is challenged by inadequate distinction between internalization and association. Imaging flow cytometry is high throughput and possesses the spatial resolution of microscopy, offering a promising route towards routine use in nanomedicine. However, the resolution of the images obtained from imaging flow cytometry is still lower than that of confocal microscopy, somewhat limiting the ability to conclusively distinguish internalized material from external material. Use of approaches with both high throughput and high spatial resolution (in parallel) should be considered as best practice for the purposes of accuracy, statistical relevance, and comparisons between studies.

- An in-depth mechanistic understanding of NP uptake will be required to improve the design of NP-based delivery platforms. The sole use of chemical compounds to inhibit or probe NP uptake pathways remains challenging because of issues such as poor specificity, cytotoxicity, cross-active chemical modalities, and varied efficacy. With proper controls, pharmacological inhibitors still provide value for simple screening of uptake pathways. However, genetic knockouts or dominant-negative proteins should be considered as complementary approaches to study uptake mechanisms. Combined chemical and genetic tools will provide data sets that more accurately represent uptake phenomena, while also allowing for closer comparison between studies. Regardless of the uptake pathway, the current understanding of how NPs induce endosomal escape lags behind and is limited by the current techniques used to detect it. Thus, new and accurate endosomal escape assays are required to understand the relationship between NP composition and endosomal escape.

- NAT are difficult to quantify if encapsulated into NP delivery carriers. The most common forms of nucleic acid measurement (i.e., UV–vis and fluorescence) provide limited insights for the characterization of NP-encapsulated NATs. Labeling and destructive methods both have drawbacks, ranging from destruction of valuable media to fundamental changes to the payload. While several multimethod approaches may be capable of providing the ratio between mass of payload and mass of substrate, the mass of payload per unit volume, and the number of payload molecules per particle, there is no single commercial solution available at present. In the interim, with no “silver bullet” method available, there is likely great benefit in a quantification regime that may allow the key dosing values to be obtained through a combination of complementary methods (i.e., individual particle size distribution measurements, total mass determination, and total payload mass/moles determination).

In summary, when considering NP therapeutic uptake, transfection efficiencies, and NAT dose, there is clear value in utilizing a combination of multiple methods to comprehensively assess features of NP systems. Importantly, our analysis of the literature indicates that the nanomedicine research community as a whole would benefit from multiparameter and multistudy reporting, which would facilitate comparison and assessment of results across studies and research groups.

Standardization of high-quality NP-mediated nonviral gene delivery studies based on multimethod, multiparameter, and multistudy reporting will enable interexperimental and interlaboratory comparisons of uptake and transfection results, ideally allowing for placing individual results obtained by one laboratory to apply in a broader context. If such a consensus can be reached, the identification of structure–function and material–biological relations of NPs could be dramatically accelerated.

## Supporting Information

File 1 Details about experimental approach and scope.
