# Supplementary material for: The steep road to nonviral nanomedicines: Frequent challenges and culprits in designing nanoparticles for gene therapy
Source: Beilstein J Nanotechnol. 2023 Mar 17;14:351–61. doi: 10.3762/bjnano.14.30 (PMC10028570; doi:10.3762/bjnano.14.30)
Supplement: File 1 — Details about experimental approach and scope. [file Beilstein_J_Nanotechnol-14-351-s001.pdf]

BEILSTEIN JOURNAL OF NANOTECHNOLOGY

Supporting Information

for

The steep road to nonviral nanomedicines: Frequent challenges and culprits in designing nanoparticles for gene therapy

Yao Yao, Yeongun Ko, Grant Grasman, Jeffery E. Raymond and Joerg Lahann

Beilstein J. Nanotechnol. **2023,** *14,* 351–361. doi:10.3762/bjnano.14.30

Details about experimental approach and scope

License and Terms: This is a supporting information file under the terms of the Creative Commons Attribution License (https://creativecommons.org/licenses/by/4.0). Please note that the reuse, redistribution and reproduction in particular requires that the author(s) and source are credited and that individual graphics may be subject to special legal provisions.
The license is subject to the *Beilstein Journal of Nanotechnology* terms and conditions: (https://www.beilstein-journals.org/bjnano/terms)

Methods: Literature search and selection process

Publication selection

The literature used for analysis was searched using PubMed NCBI® electronic database on June 27th, 2022. The advanced search function was used to identify publications including relevant terms: nanoparticles, plasmid DNA, gene, in vitro, cells, and cellular uptake or transfection (Figure S1). The search was also limited to research articles from within five years of the search date (June 2017 to June 2022) to provide an accurate reflection of current nanoparticle research practices.

The resulting 62 articles were then screened for their accessibility and eligibility. Of the 62 publications, one was excluded for being a review article, one was excluded due to inaccessibility, seven were excluded for being unrelated to nanoparticle therapy, and three were excluded due to a non-pDNA therapeutic within the nanoparticle (see Figure S1 and Figure S2 for detailed descriptions). The remaining 50 publications were included if (i) they were published in a peer-reviewed journal, (ii) they were published in English, and (iii) they were research articles involving experimental details.

Of note, the recent comment "Klein, Shannon G. et al. A prevalent neglect of environmental control in mammalian cell culture calls for best practices. *Nat. Biomed. Eng.* **2021,** *5,* 787–792" was used as the example for Figure S1 and the description of the selection process.

Data extraction

We extracted key details from Materials and Methods sections that related to the usage of imaging, flow cytometry, 3D cell culture, characterization of protein corona, and serum presence or absence during nanoparticle incubation with cells. For the serum contents of the incubation medium, we collected both qualitative and quantitative data (Figure 1e in the main article).

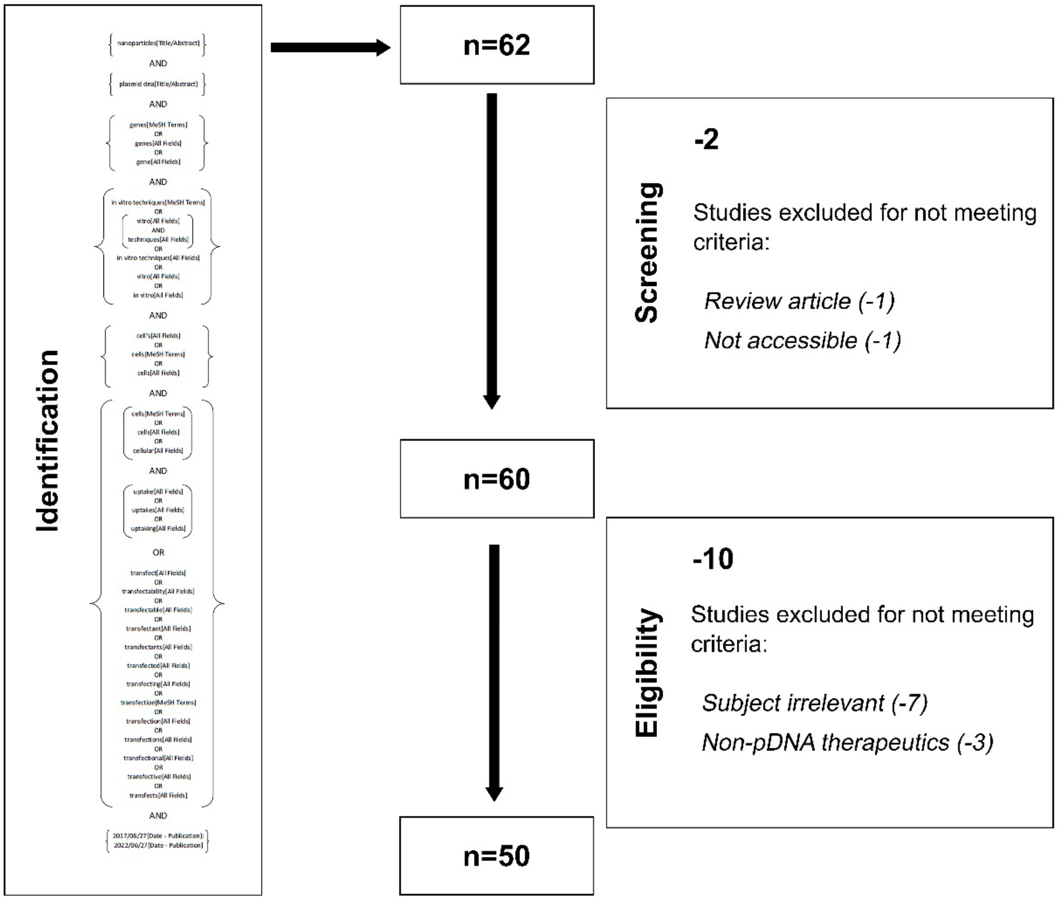

Figure S1: Flowchart of the publication selection process. The flowchart above depicts the selection process followed to find relevant publications for this Perspective article. The search term listed in the 'Identification' panel was used in the PubMed NCBI® electronic database. Publications were then excluded based on their accessibility and eligibility.

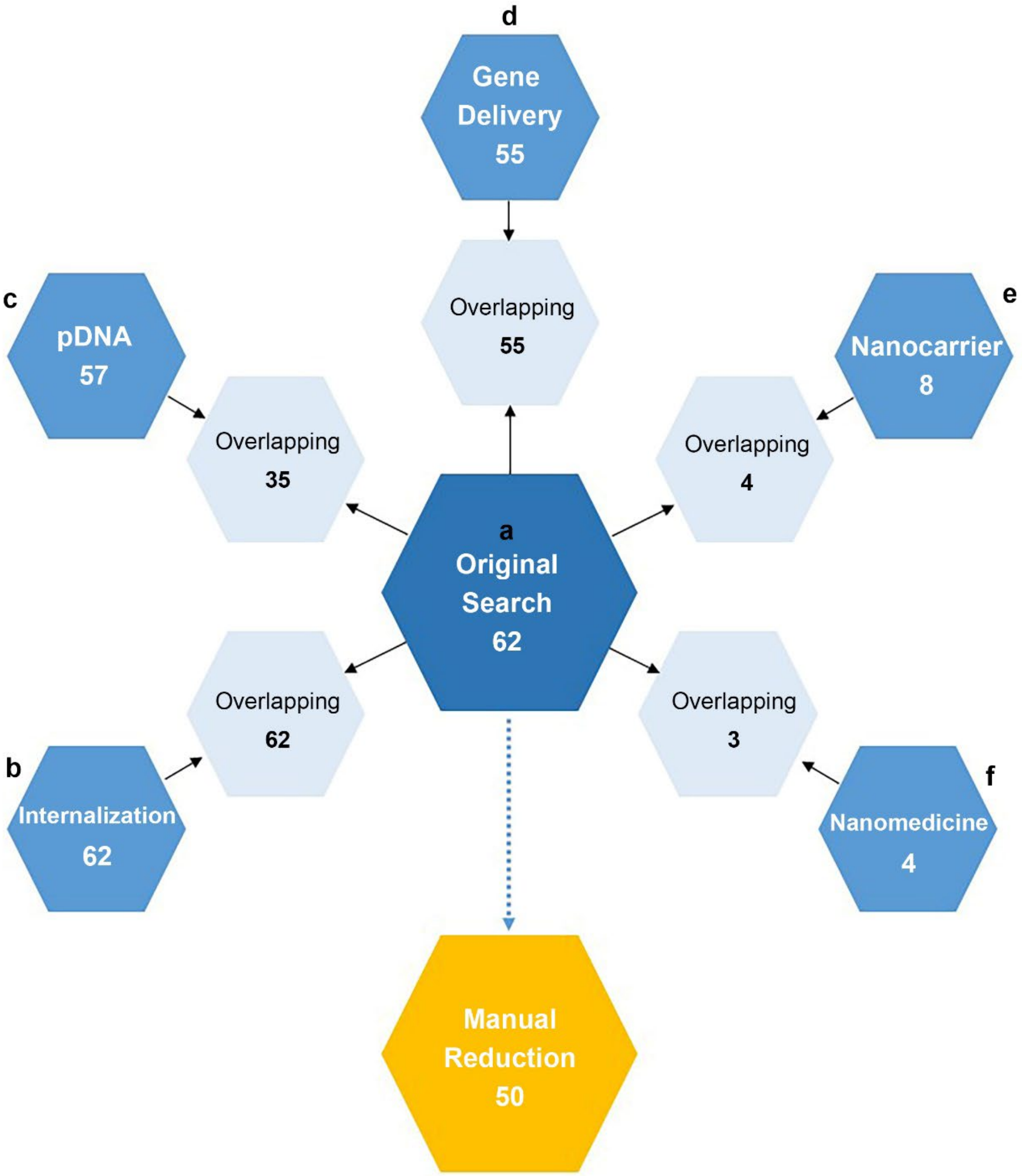

Figure S2: Search term variance analysis: variation of single-term change outcomes for the validation of the selected terms/scope. (a) Original search terms in the PubMed NCBI® electronic database: (((((nanoparticles[Title/Abstract]) AND (plasmid DNA[Title/Abstract])) AND (gene)) AND (in vitro)) AND (cells)) AND (cellular uptake OR transfection). (b) Replace "cellular uptake" with "internalization" during the literature search: (((((nanoparticles[Title/Abstract]) AND (plasmid DNA[Title/Abstract])) AND (gene)) AND (in vitro)) AND (cells)) AND (internalization OR transfection). (c) Replace "plasmid DNA" with "pDNA" during

the literature search: (((((nanoparticles[Title/Abstract]) AND (pDNA[Title/Abstract])) AND (gene)) AND (in vitro)) AND (cells)) AND (internalization OR transfection). (d) Replace “gene” with “gene delivery” during the literature search: (((((nanoparticles[Title/Abstract]) AND (plasmid DNA[Title/Abstract])) AND (gene delivery)) AND (in vitro)) AND (cells)) AND (internalization OR transfection). (e) Replace “nanoparticles” with “nanocarrier” during the literature search: (((((nanocarrier[Title/Abstract]) AND (plasmid DNA[Title/Abstract])) AND (gene delivery)) AND (in vitro)) AND (cells)) AND (internalization OR transfection). (f) Replace “nanoparticles” with “nanomedicine” during the literature search: (((((nanomedicine[Title/Abstract]) AND (plasmid DNA[Title/Abstract])) AND (gene delivery)) AND (in vitro)) AND (cells)) AND (internalization OR transfection).

Table S1: Literature list of the 50 selected papers on the topic of NP-mediated plasmid DNA delivery over the past five years (from June 2017 to June 2022).

	TITLE	JOURNAL	YEAR
**1**	Ionizable Lipid Nanoparticle-Mediated Delivery of Plasmid DNA in Cardiomyocytes	*International Journal of Nanomedicine*	2022
**2**	Targeted delivery of irinotecan and SLP2 shRNA with GRP-conjugated magnetic graphene oxide for glioblastoma treatment	*Biomaterials Science*	2022
**3**	Self-immolative polyplexes for DNA delivery	*Biomaterials Science*	2022
**4**	Lanthanide-based β-Tricalcium Phosphate Upconversion Nanoparticles as an Effective Theranostic Nonviral Vectors for Image-Guided Gene Therapy	*Nanotheranostics*	2022
**5**	Nonviral DNA Delivery System with Supramolecular PEGylation Formed by Host-Guest Pseudo-Block Copolymers	*ACS Applied Bio Materials*	2021
**6**	Relationship between phosphorylamine-modification and molecular weight on transfection efficiency of chitosan	*Carbohydrate Polymers*	2021
**7**	Optimization of Synthesis of the Amino Lipid ECO for Effective Delivery of Nucleic Acids	*Pharmaceuticals*	2021

**8**	Arf6-mediated macropinocytosis-enhanced suicide gene therapy of C16TAB-condensed Tat/pDNA nanoparticles in ovarian cancer	*Nanoscale*	2021
**9**	Characterization and Optimization of Chitosan-Coated Polybutylcyanoacrylate Nanoparticles for the Transfection-Guided Neural Differentiation of Mouse Induced Pluripotent Stem Cells	*International Journal of Molecular Sciences*	2021
**10**	Biomimetic cell membrane-coated DNA nanoparticles for gene delivery to glioblastoma	*Journal of Controlled Release*	2021
**11**	Citrate-Coated Magnetic Polyethyleneimine Composites for Plasmid DNA Delivery into Glioblastoma	*Polymers*	2021
**12**	Development and Evaluation of Solid Witepsol Nanoparticles for Gene Delivery	*Turkish Journal of Pharmaceutical Sciences*	2021
**13**	Scalable Purification of Plasmid DNA Nanoparticles by Tangential Flow Filtration for Systemic Delivery	*ACS Applied Materials & Interfaces*	2021
**14**	Cytoplasmic Trafficking of Nanoparticles Delivers Plasmid DNA for Macrophage Gene-editing	*Current Gene Therapu*	2021
**15**	Poly(ethylene glycol)-Poly(beta-amino ester)-Based Nanoparticles for Suicide Gene Therapy Enhance Brain	*ACS Applied Materials & Interfaces*	2020

	Penetration and Extend Survival in a Preclinical Human Glioblastoma Orthotopic Xenograft Model		
**16**	Efficient Delivery of Transducing Polymer Nanoparticles for Gene-Mediated Induction of Osteogenesis for Bone Regeneration	*Nanoscale*	2020
**17**	Cyclopropenium Nanoparticles and Gene Transfection in Cells	*Pharmaceutics*	2020
**18**	Multifunctional Natural Polymer Nanoparticles as Antifibrotic Gene Carriers for CKD Therapy	*Journal of the American Society of Nephrology*	2020
**19**	Electrosprayed Alginate Nanoparticles as CRISPR Plasmid DNA Delivery Carrier: Preparation, Optimization, and Characterization	*Pharmaceutics*	2020
**20**	Dicationic Amino Substituted Gemini Surfactants and their Nanoplexes: Improved Synthesis and Characterization of Transfection Efficiency and Corneal Penetration In Vitro	*Pharmaceutical Research*	2020
**21**	Arginine-modified chitosan complexed with liposome systems for plasmid DNA delivery	*Colloids and Surfaces B: Biointerfaces*	2020

**22**	In vitro and in vivo characterization of CPP and transferrin modified liposomes encapsulating pDNA	*Nanomedicine*	2020
**23**	Effect of the cagW-based gene vaccine on the immunologic properties of BALB/c mouse: an efficient candidate for Helicobacter pylori DNA vaccine	*Journal of Nanobiotechnology*	2020
**24**	Nerve Growth Factor Gene Delivery across the Blood-Brain Barrier to Reduce Beta Amyloid Accumulation in AD Mice	*Molecular Pharmaceutics*	2020
**25**	Polyethylenimine based magnetic nanoparticles mediated non-viral CRISPR/Cas9 system for genome editing	*Scientific Reports*	2020
**26**	Efficient neuronal targeting and transfection using RVG and transferrin-conjugated liposomes	*Brain Research*	2020
**27**	Peptide-Targeted Polyplexes for Aerosol-Mediated Gene Delivery to CD49f-Overexpressing Tumor Lesions in Lung	*Molecular Therapy - Nucleic Acids*	2019
**28**	Cancer gene therapy mediated by RALA/plasmid DNA vectors: Nitrogen to phosphate groups ratio (N/P) as a tool for tunable transfection efficiency and apoptosis	*Colloids and Surfaces B: Biointerfaces*	2019

**29**	Fabrication Of Gold Nanoparticles In Absence Of Surfactant As In Vitro Carrier Of Plasmid DNA	*International Journal of Nanomedicine*	2019
**30**	Development and screening of brain-targeted lipid-based nanoparticles with enhanced cell penetration and gene delivery properties	*International Journal of Nanomedicine*	2019
**31**	Zwitterion-functionalized dendrimer-entrapped gold nanoparticles for serum-enhanced gene delivery to inhibit cancer cell metastasis	*Acta Biomaterialia*	2019
**32**	Surface Coating Approach to Overcome Mucosal Entrapment of DNA Nanoparticles for Oral Gene Delivery of Glucagon-like Peptide 1	*ACS Applied Materials & Interfaces*	2019
**33**	Functionalized liposomal nanoparticles for efficient gene delivery system to neuronal cell transfection	*International Journal of Pharmaceutics*	2019
**34**	In Vitro Gene Delivery in Retinal Pigment Epithelium Cells by Plasmid DNA-Wrapped Gold Nanoparticles	*Genes*	2019
**35**	The Length of Hydrophobic Chain in Amphiphilic Polypeptides Regulates the Efficiency of Gene Delivery	*Polymers*	2018
**36**	Versatile Nanocarrier Based on Functionalized Mesoporous Silica Nanoparticles to Codeliver	*ACS Biomaterials Science and Engineering*	2019

	Osteogenic Gene and Drug for Enhanced Osteodifferentiation		
**37**	Self-assembled peptide-poloxamine nanoparticles enable in vitro and in vivo genome restoration for cystic fibrosis	*Nature Nanotechnology*	2019
**38**	Effect of the linear aliphatic amine functionalization on in vitro transfection efficiency of chitosan nanoparticles	*Carbohydrate Polymers*	2018
**39**	Optimization of the Conditions for Plasmid DNA Delivery and Transfection with Self-Assembled Hyaluronic Acid-Based Nanoparticles	*Molecular Pharmaceutics*	2018
**40**	Glycopolymers/PEI complexes as serum-tolerant vectors for enhanced gene delivery to hepatocytes	*Carbohydrate Polymers*	2018
**41**	PEGylated enhanced cell penetrating peptide nanoparticles for lung gene therapy	*Journal of Controlled Release*	2018
**42**	Hexadecylated linear PEI self-assembled nanostructures as efficient vectors for neuronal gene delivery	*Drug Delivery and Translational Research*	2018
**43**	Reactive Oxygen Species-Biodegradable Gene Carrier for the Targeting Therapy of Breast Cancer	*ACS Applied Materials & Interfaces*	2018

**44**	A laser-activated multifunctional targeted nanoagent for imaging and gene therapy in a mouse xenograft model with retinoblastoma Y79 cells	*Acta Biomaterialia*	2018
**45**	Development and characterisation of chondroitin sulfate- and hyaluronic acid-incorporated sorbitan ester nanoparticles as gene delivery systems	*European Journal of Pharmaceutics and Biopharmaceutics*	2018
**46**	Targeted, Stimuli-Responsive Delivery of Plasmid DNA and miRNAs Using a Facile Self-Assembled Supramolecular Nanoparticle System	*Biomacromolecules*	2017
**47**	Preparation, characterization, and transfection efficiency of low molecular weight polyethylenimine-based nanoparticles for delivery of the plasmid encoding CD200 gene	*International Journal of Nanomedicine*	2017
**48**	Targeting of Cellular Organelles by Fluorescent Plasmid DNA Nanoparticles	*Biomacromolecules*	2017
**49**	Superior transfection efficiency of phagocytic astrocytes by large chitosan/DNA nanoparticles	*International Journal of Biological Macromolecules*	2017
**50**	Retro-inverso d-peptide-modified hyaluronic acid/bioreducible hyperbranched poly(amido	*Acta Biomaterialia*	2017

amine)/pDNA core-shell ternary nanoparticles for the dual-targeted delivery of short hairpin RNA-encoding plasmids

Table S2: Summary of glossary.

Terminology	Definition
**Imaging**	In this paper, imaging refers to images captured by widefield fluorescence microscopy or confocal microscopy. Electron microscopy was excluded.
**Image Quantification**	Drawing quantitative conclusions from fluorescent imaging micrographs.
**2D Imaging**	In 2D imaging, a single layer of a subject is depicted. Generally, this is sufficient to make qualitative conclusions and can be used in conjunction with image analysis software to make quantitative ones.
**3D Imaging**	In a 3D imaging system, focusing can be done repeatedly on different depths of a subject. Then, image reconstruction software can assemble the multi-level image data to form a 3D reconstruction of the subject. Typically, a 3D image contains data in X, Y, and Z axes, which can provide measurements such as volume, distance, plane angle, and profile.
**Protein Corona**	When exposed to biofluids, suspended proteins may adsorb to the surface of nanoparticles and form a complex. This protein adsorption forms the so-called nanoparticle protein corona, which alters the biological properties of the nanoparticles.

**2D Cell Culture**	2D cell culture is an adherent cell culture where cells are attached and grown in a single layer on a flat and artificial surface. It typically requires periodic passaging and growth can be limited by the surface area of the artificial surfaces.
**3D Cell Culture**	3D cell culture is a culture environment that allows cells to grow and interact with surrounding extracellular framework in three dimensions. These three-dimensional cultures are usually grown in bioreactors, 3D cell colonies, or small capsules in which cells can grow into spheroids.

Discussion: Additional assessments of data extracted from literature

Protein corona

While measuring NPs in plain solvents (e.g., ddH2O or PBS) directly after preparation is a commonly used and simple approach for material characterization, it may not accurately reflect the biological performance of the NPs. The behavior of NPs under different physiological conditions, such as cell culture medium for in vitro studies and blood for in vivo studies, largely depends on their particle features in biological fluids [1]. It has been extensively acknowledged that proteins in physiological medium can bind to NPs and form a protein corona, which is typically an assembly of various proteins on the NP surface [2]. However, protein corona characterizations and studies on NP behavior in biological fluids are often overlooked when assessing newly developed gene delivery platforms. Figure 1c in the main article shows that only 10% of the reports characterized or described the protein corona. This is partly because methods for measuring NPs with nanometer or submicrometer sizes in complex fluids are still limited. In addition, polymer/DNA complex samples are typically dilute and contain large amounts of free polymer [3], which cannot be easily removed and can aggregate with serum proteins and interfere with conventional size measurements [4]. Pino et al. have proposed that improvements regarding current technical problems, including (i) insufficient NP quality, (ii) lack of precise data on NP concentration, and (iii) purification of protein–NP complexes from unbound protein, will help to expand future studies of the protein corona to new fields [5], which are highly aligned with the aims of this perspective.

Serum content

Numerous studies have explored the impact of serum components on nucleic acid-containing NPs [6,7]. When positively charged NPs first come into contact with negatively charged serum proteins, such as albumin and globulin [8], these proteins can adsorb onto the surface of the NPs. This interaction can lead to changes in the size, zeta potential, and surface characteristics of the NPs, ultimately affecting their clearance, biodistribution, endocytosis, intracellular

trafficking, and cytotoxicity [1]. Additionally, charged components in serum can disrupt the stability of nanocomplexes, potentially leading to the disassociation of nanocomplexes and further inducing the release and degradation of the nucleic acid they contain [9]. In addition, free-form polymer, which is often present in NP solutions, can also interact with negatively charged serum proteins, causing the formation of new particles. These new particles may have properties different from those of the original NPs and can potentially alter the biological effects of the NPs, such as inducing independent cellular uptake pathways [10,11].

Despite the importance of proving the biological activity of NPs in serum-containing medium (discussed above), studies conducted under both serum-containing and serum-free conditions still account for a minor proportion of studies published during the last five years (22%, Figure 1e in the main article). Previous researchers have suggested standardized procedures for screening non-viral gene delivery systems in vitro, including (i) to evaluate the effect of medium without and with serum on particle size and (ii) to replace medium with fresh medium with and without serum immediately prior to transfection [1].

3D cell culture

Monolayer cell cultures, also known as 2D cell cultures, are commonly used to evaluate the biological efficacy and cytotoxicity of NPs in vitro. However, the limited ability of 2D cultures to resemble extracellular barriers and the differences in cell phenotype between cells cultured as monolayers and cells in native tissues may contribute to poor correlations between in vitro and in vivo studies, as investigated by the Dahlman group [12]. To address this limitation and improve the predictivity of in vitro studies, researchers have turned to 3D cell culture models that better mimic in vivo conditions [10]. While 3D cell cultures are gaining popularity in the study of gene expression and other biological activities (e.g., metabolism, cell migration, and cell invasion) [13], they are not yet widely used in the NP delivery field. As shown in Figure 1d of the main article, the adoption of 3D cell culture models for NP studies is still relatively low (only 12%). This is partly due to the increased complexity and setup required compared to 2D systems, including longer cell culture times, the need for multiple growth factors, and potential risks for apoptosis. Furthermore, the determinants of cell differentiation, such as cell–cell signaling, cell–matrix signaling, tissue architecture, and mechanical forces, are specific to each tissue and are not yet well understood [14], presenting a challenge

for the development of simplified and standardized 3D culture models for NP studies.

References

1. van Gaal, E. V.; van Eijk, R.; Oosting, R. S.; Kok, R. J.; Hennink, W. E.; Crommelin, D. J.; Mastrobattista, E. *J. Controlled Release* **2011,** *154,* 218–232. doi:10.1016/j.jconrel.2011.05.001
2. Kim, W.; Ly, N. K.; He, Y.; Li, Y.; Yuan, Z.; Yeo, Y. *Adv. Drug Delivery Rev.* **2022,** 114635. doi:10.1016/j.addr.2022.114635
3. Clamme, J. P.; Azoulay, J.; Mély, Y. *Biophys. J.* **2003,** *84,* 1960–1968. doi:10.1016/S0006-3495(03)75004-8
4. van Gaal, E.; Spierenburg, G.; Hennink, W.; Crommelin, D.; Mastrobattista, E. *J. Controlled Release* **2010,** *141,* 328–338. doi:10.1016/j.jconrel.2009.09.009
5. Del Pino, P.; Pelaz, B.; Zhang, Q.; Maffre, P.; Nienhaus, G. U.; Parak, W. J. *Mater. Horiz.* **2014,** *1,* 301–313. doi:10.1039/C3MH00106G
6. Zagorovsky, K.; Chou, L. Y.; Chan, W. C. *Proc. Natl. Acad. Sci. U. S. A.* **2016,** *113,* 13600–13605. doi:10.1073/pnas.1610028113
7. Walkey, C. D.; Olsen, J. B.; Guo, H.; Emili, A.; Chan, W. C. *J. Am. Chem. Soc.* **2012,** *134,* 2139–2147. doi:10.1021/ja2084338
8. Mishra, R. K.; Ahmad, A.; Vyawahare, A.; Alam, P.; Khan, T. H.; Khan, R. *Int. J. Biol. Macromol.* **2021,** *175,* 1–18. doi:10.1016/j.ijbiomac.2021.01.152
9. Buyens, K.; Lucas, B.; Raemdonck, K.; Braeckmans, K.; Vercammen, J.; Hendrix, J.; Engelborghs, Y.; De Smedt, S. C.; Sanders, N. N. *J. Controlled Release* **2008,** *126,* 67–76. doi:10.1016/j.jconrel.2007.10.024
10. Cherng, J.-Y.; van de Wetering, P.; Talsma, H.; Crommelin, D. J.; Hennink, W. E. *Pharm. Res.* **1996,** *13,* 1038–1042. doi:10.1023/A:1016054623543
11. Miao, L.; Lin, J.; Huang, Y.; Li, L.; Delcassian, D.; Ge, Y.; Shi, Y.; Anderson, D. G. *Nat. Commun.* **2020,** *11,* 2424. doi:10.1038/s41467-020-16248-y
12. Paunovska, K.; Sago, C. D.; Monaco, C. M.; Hudson, W. H.; Castro, M. G.; Rudoltz, T. G.; Kalathoor, S.; Vanover, D. A.; Santangelo, P. J.; Ahmed, R. *Nano Lett.* **2018,** *18,* 2148–2157. doi:10.1021/acs.nanolett.8b00432
13. Abbott, A. *Nature* **2003,** *424,* 870–873. doi:10.1038/424870a
14. Goodman, T. T.; Ng, C. P.; Pun, S. H. *Bioconjugate Chem.* **2008,** *19,* 1951–1959. doi:10.1021/bc800233a
